# Relation between safe use of medicines and Clinical Pharmacy Services at Pediatric Intensive Care Units

**DOI:** 10.1016/j.rppede.2016.04.001

**Published:** 2016

**Authors:** Lucas Miyake Okumura, Daniella Matsubara da Silva, Larissa Comarella

**Affiliations:** a Hospital de Clínicas de Porto Alegre, Porto Alegre, RS, Brazil; b Hospital Infantil Waldemar Monastier, Campo Largo, PR, Brazil

**Keywords:** Critical care, Drug-related side effects and adverse reactions, Intensive Care Units, Pediatric, Pharmacy Service, Hospital, Patient safety, Medication errors

## Abstract

**Objective:**

Clinical Pharmacy Services (CPS) are considered standard of care and is endorsed by the Joint Commission International, the American Academy of Pediatrics, and the American College of Clinical Pharmacy. In Brazil, single experiences have been discreetly arising and the importance of these services to children and adolescents care has led to interesting results, but certainly are under reported. This short report aims to discuss the effect of implementing a bedside CPS at a Brazilian Pediatric Intensive Care Unit (PICU).

**Methods:**

This is a cross-sectional study conducted in a 12 bed PICU community hospital, from Campo Largo/Brazil. Subjects with<18 years old admitted to PICU were included for descriptive analysis if received a CPS intervention.

**Results:**

Of 53 patients accompanied, we detected 141 preventable drug-related problems (DRPs) which were solved within clinicians (89% acceptance of all interventions). The most common interventions performed to improve drug therapy included: preventing incompatible intravenous solutions (21%) and a composite of inadequate doses (17% due to low, high and non-optimized doses). Among the top ten medications associated with DRPs, five were antimicrobials. By analyzing the correlation between DRPs and PICU length of stay, we found that 74% of all variations on length of stay were associated with the number of DRPs.

**Conclusions:**

Adverse drug reactions due to avoidable DRPs can be prevented by CPS in a multifaceted collaboration with other health care professionals, who should attempt to use active and evidence-based strategies to reduce morbidity related to medications.

## Resumo

**Objetivo:**

Serviços de Farmácia Clínica (SFC) são considerados um padrão de atendimento à saúde e são endossados pela *Joint Commission International*, pela *American Academy of Pediatrics* e pelo *American College of Clinical Pharmacy*. No Brasil, experiências isoladas vêm surgindo discretamente e a importância desses serviços para o cuidado de crianças e adolescentes tem levado a resultados interessantes, mas que certamente são sub-relatados. Este artigo tem como objetivo discutir o efeito da implantação de um SFC à beira do leito em uma Unidade de Cuidados Intensivos Pediátricos (UCIP) brasileira.

**Métodos:**

Este é um estudo transversal, feito em uma UCIP de hospital da comunidade com 12 leitos, em Campo Largo, Brasil. Foram incluídos indivíduos com<18 anos internados em UCIP para análise descritiva, quando receberam uma intervenção do SFC.

**Resultados:**

De 53 pacientes acompanhados, foram detectados 141 Problemas Relacionados a Medicamentos (PRM) evitáveis que foram resolvidos em conjunto com os médicos (89% de aceitação de todas as intervenções). As intervenções mais comuns para melhorar a terapia medicamentosa foram: prevenção de soluções intravenosas incompatíveis (21%) e doses inadequadas (17% devido a doses baixas e altas). Entre os dez principais medicamentos associados à PRM, cinco eram antimicrobianos. Ao analisar a correlação entre o PRM e tempo de permanência na UCIP, verificamos que 74% de todas as variações no tempo de permanência eram associadas com o número de PRM.

**Conclusões:**

Reações adversas a medicamentos devido a PRM evitáveis podem ser prevenidas por SFC em uma colaboração multifacetada com outros profissionais de saúde. Tais problemas podem ser evitados por meio de estratégias ativas e baseadas em evidências para reduzir a morbidade relacionada a medicamentos.

## Introduction

The increasing number of medications being approved to adults with potential use on Pediatrics,^1^ the need to treat clinically challenging diseases, and the ethical issues surrounding pediatrics research put children and adolescents at more risks associated to medication adverse events.^2^^,^ 3 To illustrate this scenario, a nested-cohort study conducted by Bellis and colleagues^2^ demonstrated that unapproved prescriptions were associated with an augmented hazard of having an adverse event (hazard ratio 1.30, 95%CI 1.20-1.30, *p*<0.001).

To detect medication adverse reactions and to avoid preventable drug-related problems (DRPs), many accredited hospitals^4^^-^ 7 have been putting efforts to implement Clinical Pharmacy Services (CPS). Since the last decade, the multifaceted collaboration between Pediatricians, Critical Care Physicians and Clinical Pharmacists was endorsed by the American Academy of Pediatrics,^5^ American College of Clinical Pharmacy and many studies in the field.^5^^-^ 9

Despite the well-stablished importance^5^^-^ 9 of CPS to children and adolescents, in the last years, Brazil has started the implementation of single experiences around the country, especially for PICU patients, which has led to interesting but under reported results.

This study is endorsed by the evolving role of CPS in Brazil, which has been due to the recent approval of a legislation about clinical activities developed by pharmacists^10^; and the increasing interest of Latin American health institutions to get accredited.^11^ Noteworthy, Accreditation Organizations, such as the Joint Commission International, advocates that strategies to prevent medication errors, likewise pharmacists-driven clinical services, should be implemented to reduce the number of drug-related undesired events.^12^

The aim of this short report is to describe the implementation and results of a CPS directed to PICU inpatients in a Brazilian setting.

## Method

This study complies with Helsinki's Declaration and was approved by the Local Ethics Committee.

In one 12-bed community's hospital PICU located in Campo Largo, Brazil, we started the implementation of a CPS in 2012, due to accreditation processes and Clinical Director incentives to improve local health assistance. The aforementioned hospital attends all critically ill children who live approximately 200km distance from Curitiba (the biggest city in Paraná State, southern Brazil). Some of the main features of such hospital include: the presence of a computerized physician order entry, where all clinical documentations and prescriptions are electronically registered and can be remotely monitored by an online system; and, by the time of the study, one part-time pharmacist was responsible to provide CPS to inpatients (PICU and 30 bed general pediatric wards).

The CPS consisted in a systematic service dedicated to: participating in clinical rounds, elaborating institutional protocols, antiepileptic Therapeutic Drug Monitoring (TDM), reviewing each of prescribed drug dosages, indications, duration of treatments, drug interactions, relative and absolute contraindications and intravenous drug incompatibilities.

We sought to retrospectively analyze the demographics (age and sex) and clinical variables (cause of admission, comorbidities, use of vasoactive drugs, use of mechanical ventilation, use of artificial nutrition, use of antimicrobial therapy and PICU length of stay). The prevalence and types of DRPs found in such vulnerable population attended by the CPS during the implementation phase (May, October 2012) were also reported.

DRPs are defined as all situations that predisposed patients of not having optimized drug therapy, such as: intravenous solutions instability and incompatibility, wrong infusion time, high or low doses according to literature, need to adjust a dose according to renal clearance or TDM (serum concentrations of selected drugs), presence of duplicated drug therapy and wrong pharmaceutical form. Finally, we assessed the acceptance of our service by quantifying the acceptability of CPS interventions by physicians and nursing team.

Our conventional sample was calculated based on a 5% alfa, 80% power and *r*=0.50 as statistically significant correlation for this exploratory analysis, which led to 29 patients.^13^ An exploratory univariable analysis (two-tailed, Spearman rho) was performed to assess the association between DRPs and PICU length of stay. All tests were two-sided and *p*<0.05 was set as null hypothesis rejection. Descriptive statistics applied to all patients with DRPs. The aforementioned covariates were reported as median and interquartile intervals, and dichotomous variables were reported as absolute and relative numbers (%) (Table 1).

**Table 1:** Patients’ characteristics.

Characteristics	*n* (%)
*Number of included patients*	35
*Age in years*	1.50 (0.35-3.25)
*Male*	22 (63)

*Diagnostic at admission* (ICD-10)	
Infectious diseases	4 (11)
Neurologic system disorders	4 (11)
Respiratory system disorders	20 (57)
Digestive system disorders	4 (11)

*Co-morbidities during hospital stay* (International Classification of Diseases*-10*)	
Endocrine or metabolic disorders	4
Neurologic system disorders	6
Circulatory system disorders	4
Respiratory system disorders	10

*Number of drug-related problems*	141
*Mechanical ventilation*	11 (31)
*Use of artificial nutrition*	28 (80)
*Use of vasoactive drugs*	12 (34)
*Use of formulary restricted antimicrobial therapy*	15 (43)
*Intensive care lenght of stay in days*	18 (8.50-38.25)

Unless otherwise stated, all variables are expressed as absolute and/or relative (%) values. Artificial nutrition includes parenteral and enteral nutrition.All continuous variables were described as median and inter-quartile range.ICD-10, International Classification of Diseases Edition *n.* 10.

## Results

In 5 consecutive months of implementation, 53 patients were accompanied by two part-time clinical pharmacists (5h/daily dedication, except on weekends). 18 patients did not present a DRP, so they were not included in the descriptive analysis. We found 141 DRPs in 35 patients (Tables 1 and 2), who were likely to be male (63%) and were 1.50-years-old in average. Most of them were admitted due to respiratory disorders, such as acute asthma, bronchospasm and bronchiolitis-associated respiratory insufficiency. One third (31.40%) needed mechanical ventilation during PICU stay, and 34.30% used vasoactive drugs to treat hemodynamic instability.

**Table 2:** Common drug-related problems in pediatric intensive care.

Type of drug-related problems	Number (%) of drug-related problems involved with each medication					
	Meropenem	Vancomycin	Piperacillin/tazobactam	Fentanyl	Cefepime	Omeprazole
Incompatibility	6 (4)	4 (2.70)	9 (6)	2 (1.30)	6 (4)	2 (1.30)
High dose	1 (0.60)	3 (2)	-	-	-	3 (2)
Renal dose adjustment	1 (0.60)	1 (0.60)	-	1 (0.60)	1 (0.60)	-
Wrong infusion time	-	1 (0.60)	-	3 (2)	-	-
Low dose	1 (0.60)	1 (0.60)	-	2 (1.30)	-	-
Therapeutic drug monitoring dose adjustment	-	-	-	-	-	-
Duplicated drugs	-	-	-	-	-	1 (0.60)
Wrong pharmaceutical form	1 (0.60)	-	-	-	-	1 (0.60)
Total (%)	10 (6.80)	10 (6.80)	9 (6)	8 (5.30)	7 (4.70)	7 (4.70)
Type of drug-related problems	Number (%) of drug-related problems involved with each medication					Other
	Oseltamyvir	Captopril		Methyprednisolone	Phenobarbital	
Incompatibility	-	-		1 (0.60)	-	24 (17)
High dose	-	1 (0.60)		3 (2)	1 (0.60)	10 (8)
Renal dose adjustment	-	2 (1.30)		-	-	10 (8)
Wrong infusion time	-	-		-	-	4 (3)
Low dose	-	1 (0.60)		-	-	7 (5)
Therapeutic drug monitoring dose adjustment	-	-		-	2 (1.30)	7 (5)
Duplicated drugs	-	-		-	-	4 (3)
Wrong pharmaceutical form	5 (3.30)	-		-	-	4 (3)
Total (%)	5 (3.30)	4 (2.70)		4 (2.70)	3 (2)	74 (53)

Selected drugs accounts for 67 (47%) from 141 drug-related problems (DRP) found by pharmacists. Stability, compatibility and dose were common problems identified by clinical pharmacists. “Others” column refers to drugs that were less common. Only drugs with more than 4 DRPs were reported.

**Figure 1:**
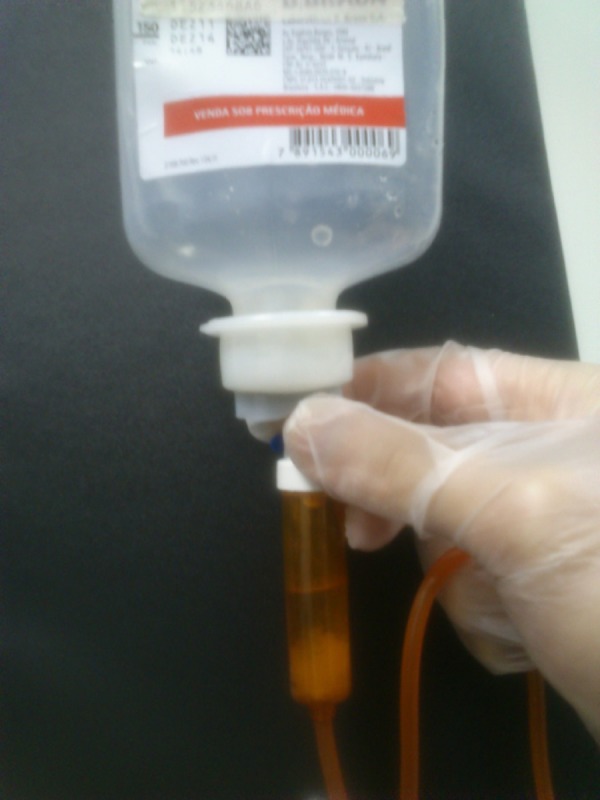
Incompatibility problems avoided by Clinical Pharmacy Services.

Out of the 141 DRPs detected by CPS, the most common interventions performed to improve drug therapy were: preventing incompatible intravenous solutions (21%) and a composite of inadequate doses (17% due to low, high and non-optimized doses) (Fig. 1). Among the top ten medications associated with DRPs, five were antimicrobials: meropenem, vancomycin, piperacillin and tazobactan, cefepime and oseltamyvir (Table 2).

By analyzing the Spearman-rho correlation between DRPs and PICU length of stay, we found that 74% of all variations on PICU length of stay were associated with the detected DRPs.

## Discussion

In our sample, the implementation of a CPS directed at PICU inpatients has shown the value of such services on detecting and solving DRPs, which were at most preventable situations that could lead to unnecessary morbidity.

Through an average 33 days of PICU stay (95%CI 20.22-46.38), we found that each patient could be exposed to as much as 2.6 DRPs, and interventions toward solving them were highly accepted by medical and nursing team (89% acceptability rate). Such acceptance of interventions by PICU team was consistently high, as already demonstrated before.^8^^,^ 11 ^,^ 12 The message behind these findings stands for a good CPS implementation process, which had as determinants of success: the institutional support and communication between hospital's pharmacy manager, clinical director, PICU nurses and infectious disease team.

Still on DRPs detected, as shown in Table 2, stability and compatibility problems were commonly seem with piperacillin and tazobactam. Wrong infusion time was commonly detected with fentanyl, and duplicated pharmacotherapy was more prevalent with omeprazole (intravenous and oral routes prescribed). Sub-therapeutic doses of phenobarbital were corrected by pharmacists, either by literature-based information or by TDM.

Few studies^8^^,^ 14 ^-^ 17 were already published in PICU settings, but none comes from Latin American countries. A single randomized controlled trial^8^ assessed the effectiveness of CPS in reducing inpatient length of stay. An observational study conducted in French-speaking countries described 966 interventions done to solve DRP in 270 patients, through 6 months of CPS implementation.^14^ Other researches had also showed positive results. A cohort study conducted in United States included 1120 patients and found that half of patients were exposed to medication errors. They found that 28% of all problems detected were related to dose, and other 18% with wrong route of administration.^15^ In United Kingdom, antibiotics and inotropes were reported to be the top drugs associated with medications errors.^16^ Our study showed similar results by having meropenen, piperacillin and tazobactam, vancomycin, cefepime and oseltamyvir as part the top ten medications associated to DRP.

Unfortunately, some studies^16^ did not specify the details of the medication errors detected, which are indispensable for PICU pharmacists. To overcome such lack of descriptive information, our study identified that weight variation, acute kidney injury and TDM led to dose adjustment interventions, namely: vancomycin, captopril and phenobarbital.

Our research was not free of limitations and some of them deserve special attention. At first, confounders are inherent to cross-sectional studies and some of the assumptions made in this manuscript should be further investigated in larger prospective cohorts. At second, because it was not part of our first objective, we did not provide a descriptive characterization of all drugs used in our PICU. On the other hand, we focused on: (a) clinical description of the population, which is important to physicians and clinical pharmacists; (b) the main DRPs found, which is of special interest to other settings that aim to implement such services. At third, our casuistic comprised children and adolescents, but not neonates, who are subject of higher risks of adverse drug reactions.^18^ Herein, when interpreting our results, external validity of our data should be carefully interpreted, given that we did not attend trauma, large surgeries, and neoplasms. In addition, the univariate analysis should be interpreted carefully, due to our study's limitations. On the other hand, it reinforces^6^^,^ 8 ^,^ 9 the importance of monitoring long term critically ill inpatients, given that DRPs may be more prevalent in this population, which could lead to undesired drug-related events. Lastly, data collection is a common drawback from retrospective studies. We sought to reduce such problems by having three post-graduated pharmacists in this activity, who consulted each other when discrepancies/inconsistencies were found.

Every ten patients admitted to PICU, six had a DRPs detected by CPS and five received an intervention to optimize drug therapy. PICU setting has a high prevalence of compatibility and stability DRPs (Table 2), and dose adjustments should be promptly assessed especially on inadequate therapeutic drug serum concentrations, weight changes and other risk factors that may change drug distribution and excretion, such as acute kidney injury. Based on our implementation experience, CPS might be a feasible technology that improve infants, children and adolescents care. Pediatricians' and stakeholders should attempt to prevent DRPs by using active and evidence-based strategies to reduce avoidable morbidity-related to medications.^4^^-^ 6 ^,^ 8 ^,^ 9
